# Observations on the Puerperal Fevers Which Occurred at the Maternité of Paris in 1829; on the Various Modes of Treatment That Were Adopted, and Particularly on Mercurials, Emetics, and Bloodletting

**Published:** 1830-09

**Authors:** M. Tonnelle


### PUERPERAL FEVERS.

*Observations on the Puerperal Fevers which occurred at the Maternité of Paris in* 1829 *; on the various Modes of Treatment that were adopted, and particularly on Mercurials, Emetics, and Bloodletting.* By M. Tonnelle. (Second Article.)

Case VII. *Puerperal Fever, with Peritonitis and Uterine Phlebitis; Gangrene of the Lungs; Softening of the Mucous Membrane of the Stomach; Abscess in the Leg.*

Marie Bapt..., æt. twenty-three, of good health, complained, the second day after a favorable delivery, of acute pains in the hypogastrium and fever. Forty leeches were immediately applied to the part in pain, with great relief; but on the next day the pains returned, with copious diarrhœa and frequent vomiting. Lochia suppressed, and the breasts flaccid. Sixty leeches were now applied to the abdomen, and forty more in the evening.

Third day. Lochial discharge returned, and the patient no longer suffering.

Fourth day. She was restless, and frequently fainted.

Fifth. Delirium, and complains of pain in the leg, which was swollen on the anterior part.

Sixth. She expectorated some fetid sanguineous matter; stools were passed involuntarily; she sank into a state of insensibility, and died the following day.

From the third day, two ounces of mercurial ointment had been rubbed in every twenty-four hours.

*Dissection.* In the abdominal cavity there was a considerable quantity of puriform serum, mingled with false membranes. Cervix uteri and ligamenta lata infiltrated with pus, and most of the uterine veins were filled with the same liquid. Extremity of the fallopian tubes thickened; ovaria swollen and softened. In the centre of the right lung there was a gangrenous cavity, about three or four inches in extent, which was half filled with black and fetid shreds, and a thick matter of the same colour and appearance. The mucous membrane of the stomach was softened in a great part of its extent, and entirely destroyed around the cardiac orifice. Upon the surface of the colon were some spots of ulceration. There was also a peculiar suppuration in the leg. The deep-seated muscles were infiltrated and soaked through with pus, for the space of three or four inches. Around this deposition of matter, and in various parts of the leg there were several small collections of matter, which appeared to be formed in the middle of the muscles. The muscles, however, retained their natural colour: the interstitial and subcutaneous cellular tissue neither presented that redness or serous infiltration which usually accompanies deep-seated inflammations.

In this case, as in the preceding,* the various morbid appearances that were detected could only be attributed to the influence of some general cause, which gave rise to the whole. The number of the lesions, their rapid and insidious progress, and their destructive nature, give to them a peculiar character, which is not presented by the ordinary forms of inflammation; but which may be explained by the deleterious influence of vitiated blood upon all the organs. The suppuration in the muscles of the leg deserves particular attention; and, for the purpose of illustrating more clearly these kind of abscesses, we shall record a few other facts.

Case VIII. *Puerperal Fever, with Uterine Phlebitis; Purulent Collections in the Psoæ, Iliac, and Triceps, &c. Muscles.*

G...., æt. thirty-seven, of good constitution, was attacked, on the third day after a favorable labour, with all the symptoms of intense puerperal fever. By a free use of leeches, the disease was much relieved towards the eighth day. The febrile symptoms and pains in the abdomen then returned with great severity. The patient soon became agitated and delirious. A state of profound coma succeeded, which was occasionally interrupted by cries and moans. She died on the fifth day of the relapse.

Mercurial frictions in large quantities, calomel, and mercurial clysters, were the means employed during the latter period of the disease.

*Dissection, twenty-four hours post mortem.* In addition to the ordinary appearances of intense uterine phlebitis which extended through all the veins of the organ, the following alterations were observed: The psoæ muscles of both sides contained several large purulent collections: the matter was situated in the centre of the muscles, in well circumscribed deposits. The fleshy fibres in contact with the pus were of a greyish colour, and softened; but beyond this they presented their natural colour and consistence. There were several similar deposits of matter in the right iliac muscle, and one of a smaller size in the substance of the triceps of the thigh. The brain and every other part healthy.

* Vide our Number for July, p. 19.

Case IX. ***Puerperal Fever, with Uterine Phlebitis. Abscesses in the Muscles of the Legs, Thigh, Forearm, and Knee-joint.***

E. Hain. . ., æt. twenty-three, delicate and nervous, was attacked with puerperal fever the fourth day after her first labour. An emetic of ipecacuanha, and fifty leeches to the hypogastric region, quickly dispelled the symptoms; but, on the fourteenth day, the patient, who was already convalescent, felt very great anxiety, the cause of which could not be ascertained.

Fifteenth day. Violent fever, frequent vomiting, deep-seated and intense pain in the left hypogastric and iliac regions. Forty leeches were applied with decided, although but temporary, benefit. A series of severe symptoms quickly arose: Slight delirium, a stupid and intoxicated look, occasional agitation, tongue clean, small and frequent pulse, copious and fetid diarrhœa, deep-seated pain in the hypogastric region, and nausea.

Towards the end of the attack, she complained of pain in the legs and forearms, and, although there was neither external redness, shining of the integuments, nor swelling of the soft parts, M. Desormeaux predicated the existence of purulent collections in the substance of the muscles. The patient soon died, and his opinion was verified upon dissection.

Thick well-formed pus was infiltrated in the substance of the deep-seated muscles of both legs. The muscular fibres in contact with the matter were grey and soft, but immediately beyond they suddenly appeared of their natural colour and consistence. Several small, isolated, and well-circumscribed collections, about the size of an almond, were seen in the solar and anterior tibial muscles, and in the middle of the thigh. Precisely the same appearances were detected in the forearm. In the left knee-joint there was also a certain quantity of well-formed pus. The synovial membrane retained its natural delicacy of structure, as well as its polish and transparency. The veins of the uterus were filled with thick laudable pus; their internal membrane was of a greyish colour, rugged and unequal. The left ovarium was converted into a cavity, half filled with pus, which adhered to the rectum, and opened into the intestine. Peritoneum natural. Several red spots were seen on the stomach. Other parts healthy.

The tenth case was one of puerperal fever, with suppuration in the veins and lymphatics of the uterus. There were numerous abscesses in the muscles, and a collection of matter in the knee-joint.

Cases of this kind are by no means rare, and some examples are contained in the works of Leake, Doublet, &c. Many are recorded in recent theses, and in the memoir by M. Dance. M. Desormeaux witnessed many instances of this sort. It is evident that the purulent collections above described differ essentially from ordinary abscesses. The latter but rarely occupy the substance of muscles: they are situated in the cellular intervals which separate the muscles or their different fasciculi; or around the bones, or in the subcutaneous cellular tissue, &c. They form in a more or less rapid manner, but always gradually and regularly. They are usually accompanied by violent pain, and even in the deepest-seated phlegmons the integuments are of a purplish colour, or are tense and shining. Common inflammation is not confined to precise and well-defined limits, so as to form exactly circumscribed collections of matter, like those above described: it is generally diffused, and not equally violent in all the parts it occupies. In every case it disappears insensibly by a sort of gradual union with the surrounding parts. The purulent collections which existed in the preceding cases constantly occupied the centre of the muscles, and not their cellular intervals. This fact we shall afterwards endeavour to explain.

The accompanying pain was not severe, and such as the mechanical distention of the parts would account for. Neither the aspect nor colour of the integuments were altered: the muscles, indeed, were softened, but only where they were steeped in matter; beyond this point they had a natural appearance. Frequently, indeed, pus was seen in small insulated collections in the midst of muscles which appeared perfectly healthy. The number of these purulent deposits, and the rapidity of their formation in different and distant parts, are also remarkable. Do we ever see common inflammation thus occupy many different points, in each of the same violence, and terminating every where in the same manner? If, at the same time, there exists in the economy a general, manifest, and even palpable cause, which will explain all these various effects; if we detect pus in the uterine vessels, from whence it is conveyed through all the circulating channels with the blood which serves as its vehicle; if characteristic symptoms show, almost to a certainty, that pus has been conveyed to the various organs; and if these symptoms constantly precede, and enable us to foretel the developement of the abscesses in question, we have surely sufficient grounds for believing that the purulent collections of the lungs, liver, brain, &c. observed in such cases, are not the effects of ordinary inflammation, but the result of a peculiar process, or a simple deposit of matter in the substance of the muscular tissue.

It may possibly be objected that this hypothesis, however admissible it may be as far as regards the lungs and liver, which are the principal centres of the venous circulation, cannot apply to the muscles, which receive but a limited supply of blood. But there are many veins distributed to these organs; and, besides, experiments have been made which strengthen the above supposition. M. Cruveilhier injected mercury into the veins of animals, and found it extravasated in the muscles, as well as in the principal viscera. In some of the above cases, indeed, the purulent deposits were confined to the muscles, while there were no traces of such appearances in any other part of the body. For an answer to this objection, we must again refer to M. Cruveilhier. "I have frequently," says he, "seen the mercury traverse the capillary system of the lungs, and fix itself especially in the substance of muscles and in the serous cavities. It may be difficult to explain these different phenomena, but the facts are undoubted, and to them speculation must yield. It appears, then, rational to admit that these purulent collections in the muscles and articulations, which are so often connected with phlebitis, are the direct and immediate effect of an absorption of matter, and of its mixture with the blood. We may conceive them to arise in the following manner.

Sometimes a certain number of purulent particles being fixed in the substance of the muscles, or deposed upon the surface of serous membranes, produce inflammatory action, and become the centre of a circumscribed inflammation, which terminates rapidly in suppuration: in this manner the globules of mercury acted in the experiments we have just referred to. Sometimes, on the contrary, the pus appears to be deposited in the muscles without any local process. Although it may be difficult to admit this fact, it appears almost impossible to explain in any other manner the formation of some of the small isolated depositions of pus, which were detected in the substance of muscles in other respects healthy, and particularly those purulent collections which M. Desormeaux has sometimes seen form immediately under the skin with the greatest rapidity, and without the least appearance of any inflammatory process.

It is objected that the laws of physiology are in direct opposition to such facts; but it is forgotten that the act of nutrition itself is but the result of a similar deposition, which differs only by its constancy and regularity. If the vessels constantly deposit in different organs certain particles which are destined to repair their tissue, why should they not, in the same manner, unload themselves of any heterogeneous principles they may contain.

In all the preceding cases, the typhoid symptoms, the invariable effect of uterine phlebitis and the absorption of pus, terminated rapidly in the death of the patient. Such is not always the case. Frequently the disease is less severe, and gradually disappears under judicious treatment, and perfect health is restored.* It is a fact which may be deduced from all the previous cases, that the second period of uterine phlebitis is in fact typhous fever. It has been proved, indeed, by the experiments of Gaspard, Dupuy, &c. that all the symptoms of typhous fever may be produced at will, by injecting pus or putrid matter into the veins of animals. The typhoid fevers which occur after labour do not always depend upon the same cause: the disease is sometimes primarily developed after labour, and neither the symptoms observed during life nor the dissection after death shows any evidence of uterine phlebitis, inflammation of the womb, or any other morbid alteration.

Case XIII. ***Primary Typhoid Fever after difficult Labour, with abundant Hemorrhage.***

Garn..., æt. thirty, of good health, entered the Maternité, the 2d of August, 1819, and was delivered some days afterwards of her first child. The labour was long and very painful. From inertia of the uterus, the delivery was accomplished by the forceps. Profuse hemorrhage followed, which was arrested with difficulty by injections and cold applications. On the following day the lochial discharge was abundant. The patient was pale and much exhausted: she complained of deep-seated pain in the hypogastric region, and it was supposed that inflammation of the womb was arising. M. Desormeaux ordered fifty leeches to the abdomen, and a hip bath.

Third day. Fever, heat, and thirst. Lochia continued; no pain in the abdomen.

* M. Tonnellé here details two cases, both of which he presumes were instances of uterine phlebitis followed by typhous fever. The patients recovered. As the proof of their pathological character appears very unsatisfactory, we have not translated his report of them.—Ed.

Fifth day. Large sloughs were found on the labia ; diarrhœa ; patient much depressed.

Eighth day. Great agitation; delirium; involuntary discharge of fetid stools. Tongue dry; pulse quick and irregular. The features and general aspect of the patient expressed a state of profound prostration.

On the eleventh day, she died.

The treatment consisted at first of rice drink; and subsequently decoction of bark ; and pills of extract of bark and camphor were given.

*Dissection, thirty hours after death.* The body was already partially decomposed. The labiæ were converted into a thick deep slough. Uterus large, and its tissue flaccid, but perfectly healthy, as well as its vessels. Stomach and intestines perfectly white, without any alteration of their substance or consistence. The Peyerian glands were scarcely perceptible. Lungs gorged with blood ; heart soft and shrunk, its cavities filled with fluid blood. Brain very soft and pale. Every other part was healthy.

Case XIV. *Typhoid Fever after Labour. Gangrenous diathesis; disorganization of the stomach.*

Berge..., ætat. twenty-four, of a nervous and irritable constitution, suffered, at the commencement of her first pregnancy, from vomiting and pains in the stomach. She was bled, and leeches were frequently applied. At the third month of pregnancy, she was attacked with an inflammatory swelling of the knee, and was admitted into the Hôtel Dieu. In five months she was discharged nearly cured, but much exhausted by severe suffering, debilitating treatment, and her long residence in the hospital. She soon entered the Maternité, and passed through her labour very favorably. Eight days after, she was attacked with fever and diarrhœa. The tongue soon became dry, and was covered with a thick brown coat; features much altered, mind confused. There were soon constant delirium, a wild look, trembling of the limbs, involuntary stools; small, quick, and irregular pulse. At the same time large and deep sloughs formed upon various parts of the body, upon nearly the whole of the breasts, the sacrum, the fore part of the thighs, and both heels. Towards the close of her existence, there were nausea, feeble attempts to cough, and profuse viscid sweats. She died on the tenth day of the disease, after a long and painful agony.

Acidulated drinks, extract of bark with aromatics, blisters to the legs, which were dressed with powder of bark and spirits of camphor, constituted the treatment.

*Dissection, twenty hours post mortem.* Nearly the whole surface of the breasts and sacrum were in a state of slough. The labia, or great part of the integuments of the thigh, and both heels, were in a similar condition. The stomach had a large opening upon the cardiac extremity: the edges of this orifice were soft and fringy, and of a blackish colour, which was gradually lost in the white tint of the other parts of the organ. There was no trace of inflammation, either around the perforation or in any other portion of the stomach. Peritoneum, uterus, and its appendages, perfectly healthy. Every other part was examined, but there was no alteration of consequence detected.

These two last cases were different in form, but essentially the same. Both of them were examples of very strongly marked and severe typhoid fever; but if they are compared with the previous cases, we shall immediately be struck with a very important distinction. In the latter, the typhoid affection was consecutive to suppuration of the uterus or its vessels, and to the transport of pus into the circulating channels. In the instances just related, on the contrary, the typhoid disease was developed primarily and independently of any appreciable alteration. The progress of the symptoms, and the state of the organs, can leave no doubt upon this subject. The first of the two last-named patients experienced, it is true, the day after her labour, some pain in the epigastrium; but these pains gradually decreased, and they had no influence on the pulse, which remained undisturbed, nor on the lochial discharge, so that they could only with reason be attributed to fatigue of the uterus, the consequence of a long and difficult labour, and the use of the forceps. In the case of the other woman, there was no abdominal pain, and it was, besides, at the very commencement that all the typhoid symptoms occurred. Lastly, in both these instances, the most careful dissection detected no morbid appearances, either in the peritoneum, uterus, or its appendages. The state of these organs corresponded, therefore, with the progress of the symptoms.

There can be no doubt that many of the cases of "putrid puerperal fever" related by different writers, were instances of uterine phlebitis, although the nature of the malady was not known; but we must also remember that many cases of the disease are primitive affections, very nearly similar to the last we have related. It is a question worth examining, whether the developement of such diseases is connected directly and immediately with the puerperal state, or whether they are fortuitous and purely accidental. It is certain that the ancients attributed more influence to the puerperal state than it really possesses. They were struck with the undeniable peculiarity of this condition of the female, and they fancied that every disease which arose after labour, was specially influenced by it: the term puerperal was therefore applied indiscriminately to a variety of very dissimilar maladies; and, as happens in the present day with respect to inflammation, from generalizing too much, confusion and uncertainty were produced. More recently, the investigations of Pinel and Bichat, and different physicians of our own day, by limiting the term puerperal fever to inflammation of the peritoneum and uterus, have excluded many different affections that were formerly included in it. Although parturition is a natural act, it cannot be denied that, even in the most simple cases, it makes a violent impression upon the whole economy, and gives a general shock, from which arise some of the features of typhoid fever. "The pains of labour," says Desormeaux, "powerfully affect the nervous system, and produce considerable excitement. . . . The pulse becomes hard and frequent, the heat of the body is increased, the face is flushed, the lips and tongue are dry; there is, in fact, a universal and great agitation. . . . At a later period the agitation is still greater; the parturient efforts are accompanied by convulsive trembling, and sometimes, even in ordinary cases, the intellectual functions are disturbed."

This temporary disturbance can no more be regarded as a pathologic condition, than that which arises from passion or from any excessive mental emotion. But, if this violent process of pain is to be supported by a woman who is already weakened by disease, low diet, or the air of an hospital; or if it be prolonged much beyond its natural duration, and abundant hemorrhage should occur, and increase the exhaustion produced by the pains of labour, as was the case in the two last-related instances, we think that these united causes will be sufficient to explain the developement of typhoid fever." When a laborious or preternatural labour, says Dr. West, has been too long procrastinated, and the power of the uterus is destroyed by useless efforts, and its parietes weakened and contused, we observe rather the indicative signs of death, than the symptoms of a disease: the eye is dull and dry, the features are altered, voice extinct, skin of a leaden hue, and covered with cold sweat; the pulse is very feeble; the pains soon cease; the excitability of the patient is exhausted; the intellectual faculties are disturbed; a paroxysm of fever sometimes succeeds the cold sweats, and death follows in twenty-four or forty-eight hours."

We have in this sketch the characters of typhoid fever in its highest degree, and the correlation between the symptoms and the process of labour is evident.

Were we to extend the argument beyond the circle of puerperal diseases, we could easily support our opinion by a multitude of analogous facts. The suffering from surgical operations or extensive burns is sometimes followed, like that of labour, by typhoid symptoms. But we can only hint at these analogies, without, upon the present occasion, entering more at length into their consideration.

(To be continued.)

---

